# SARS-CoV-2 Transmission Associated with High School Wrestling Tournaments — Florida, December 2020–January 2021

**DOI:** 10.15585/mmwr.mm7004e4

**Published:** 2021-01-29

**Authors:** Christine Atherstone, Molly Siegel, Emily Schmitt-Matzen, Scott Sjoblom, Joy Jackson, Carina Blackmore, John Neatherlin

**Affiliations:** ^1^CDC COVID-19 Response Team; ^2^Epidemic Intelligence Service, CDC; ^3^Florida Department of Health in Polk County, Bartow, Florida; ^4^Florida Department of Health.

*On January 26, 2021, this report was posted online as an *MMWR *Early Release.*

On December 7, 2020, local public health officials in Florida county A were notified of a person with an antigen-positive SARS-CoV-2 test* result who had attended two high school wrestling tournaments held in the county on December 4 and 5. The tournaments included 10 participating high schools from three counties. The host school (school A in county A) participated in the tournaments on both days; five high school teams from two counties participated the first day only; four additional high school teams from the three counties participated the second day. A total of 130 wrestlers, coaches, and referees attended the tournaments (Table). During December 8–9, 13 wrestlers from school A received positive SARS-CoV-2 test results (Figure), including nine who were symptomatic, two who were asymptomatic, and two for whom symptom status at time of specimen collection was unknown. Local public health officials in the three counties initiated an investigation^†^ and tested specimens from an additional 40 attendees from nine of the 10 participating schools. A total of 54 (41.5%) of the 130 tournament attendees received testing, and 38 cases of SARS-CoV-2 infection were identified; the minimum attack rate was 30.2% (38 of 126^§^), and 70.4% (38 of 54) of tests had a positive result. Among contacts of the 38 COVID-19 patients, 446 were determined by investigators to meet the CDC definition of a close contact,^¶^ including 62 who were household contacts and 384 who were in-school contacts (classmates, teachers, noncompeting wrestling team members, and other school athletic team members). Among these 446 contacts, five had received a diagnosis of COVID-19 during June–November and were excluded from attack rate calculations. Among 95 (21.3%) contacts who received SARS-CoV-2 testing, 41 (43.2%) received a positive test result (minimum attack rate = 9.3% [41 of 441]); 21 (51.2%) persons with positive test results were symptomatic, eight (19.5%) were asymptomatic, and symptom status for 12 (29.3%) was unknown at the time of specimen collection. Among contacts, attack rates were highest among household members (30.0%) and wrestling team members who did not attend the tournament (20.3%), as were the percentages of positive test results (60.0% among household members and 54.2% among team members). Among all contacts, the odds of receiving a positive test result were highest among household contacts (odds ratio = 2.7; 95% confidence interval = 1.2–6.0). Local health authorities reported the death of one adult contact aged >50 years.

**TABLE:** Characteristics of persons with COVID-19 associated with high school wrestling tournaments — Florida, December 2020–January 2021

Characteristic	No. of persons (%)			Attack rate, % (no. positive/no. susceptible)^†^
	Total	Received testing	Had positive test results*	
**Tournament attendees**				
All attendees	130 (100.0)	54 (41.5)	38 (70.4)	30.2 (38/126)
Wrestlers	116 (89.2)	44 (37.9)	31 (70.5)	27.4 (31/113)
Coaches	6 (4.6)	5 (83.3)	3 (60.0)	60.0 (3/5)
Referees	5 (3.8)	2 (40.0)	1 (50.0)	20.0 (1/5)
Other^§^	3 (2.3)	3 (100.0)	3 (100.0)	100.0 (3/3)
**Contacts**				
All contacts	446 (100.0)	95 (21.3)	41 (43.2)	9.3 (41/441)
Household	62 (13.9)	30 (48.4)	18 (60.0)	30.0 (18/60)
Classmates and teachers^¶^	168 (37.7)	30 (17.9)	10 (33.3)	6.0 (10/166)
Team members not attending tournaments^¶^	64 (14.3)	24 (37.5)	13 (54.2)	20.3 (13/64)
Other school athletic members^¶^	152 (34.1)	11 (7.2)	0 (—)	— (0/151)
Age group of contacts, yrs**				
0–13	18 (4.0)	8 (44.4)	5 (62.5)	27.8 (5/18)
14–18	384 (86.1)	71 (18.5)	27 (38.0)	7.1 (27/380)
19–24	8 (1.8)	2 (25.0)	1 (50.0)	12.5 (1/8)
25–44	22 (4.9)	7 (31.8)	3 (42.9)	14.3 (3/21)
45–61	12 (2.7)	7 (58.3)	5 (71.4)	41.7 (5/12)

**Abbreviation:** COVID-19 = coronavirus disease 2019.

* Among those receiving testing.

^†^ Four tournament attendees and five contacts received positive test results during June–November 2020 and were not included in the attack rate calculation.

^§^ “Other” category includes a nonwrestling high school athletic coach and two students who were not wrestlers.

^¶^ Within-school contacts.

** Ages of two contacts were unknown.

**FIGURE:**
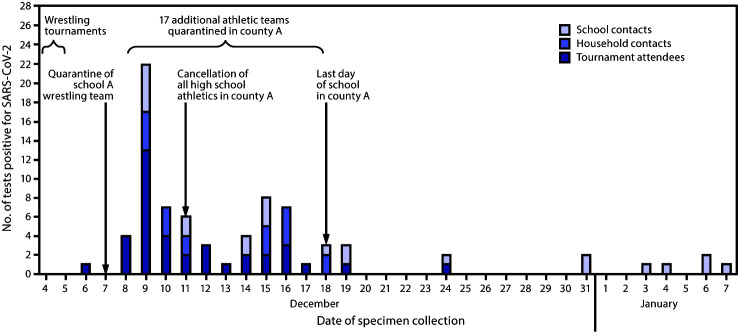
SARS-CoV-2 tests with positive results among attendees of high school wrestling tournaments and their contacts, by specimen collection date — Florida, December 2020–January 2021

An estimated 1,700 in-person school days were lost as a consequence of isolation and quarantine of patients and contacts during this COVID-19 outbreak.** The number of in-person school days lost would likely have been higher had the outbreak not occurred toward the end of the fall 2020 semester. In addition, this outbreak resulted in the suspension of all winter indoor and outdoor high school athletics in county A, affecting approximately 1,500 students.

The American Academy of Pediatrics interim guidance for return to sports specifically recommends against mask wearing during wrestling because of the choking hazard that face coverings could pose (*1*). In October, local public health and school officials in county A established COVID-19 mitigation guidelines specific to wrestling for practices, matches, and tournaments, including mask wearing and physical distancing (at least 6 feet) when not actively wrestling, symptom screening, and disinfection of space and equipment. However, it is not feasible to maintain physical distancing and universal mask wearing during practice and competition for high-contact sports such as wrestling.

At the time of the tournament, the 14-day cumulative COVID-19 incidence in county A, home to seven of the 10 participating high school teams, was 363 per 100,000 persons; 7.7% of tests for SARS-CoV-2 had positive results (*2*). The incidence in county A placed the community in the highest category for transmission of SARS-CoV-2.^††^ CDC guidance provides community transmission level thresholds for school decision-makers that should be applied to school athletics and related social gatherings. High-contact school athletic activities for which mask wearing and physical distancing are not possible should be postponed during periods with substantial or high levels of SARS-CoV-2 community transmission (*3*). Outbreaks among athletes participating in high contact sports can impact in-person learning for all students and increase risk for secondary in-school and community transmission with potentially severe outcomes including death (*4*).

## Footnotes

* County A used the Abbott BinaxNOW COVID-19 Ag Card (BinaxNOW) rapid antigen test in testing for symptomatic persons, with follow-up reverse transcription–polymerase chain reaction (RT-PCR) testing if the antigen test was negative, and RT-PCR testing for all asymptomatic persons; results were considered positive if either the BinaxNOW or RT-PCR test result was positive. Wrestling tournament attendees and contacts were encouraged to seek COVID-19 testing services regardless of symptoms, but the decision to be tested was left to each person.

† This activity was reviewed by CDC and was conducted consistent with applicable federal law and CDC policy: 45 C.F.R. part 46.102(l)(2), 21 C.F.R. part 56; 42 U.S.C. Sect. 241(d); 5 U.S.C. Sect. 552a; 44 U.S.C. Sect. 3501 et seq.

§ Four tournament attendees received positive test results during June–November 2020 and were not included in the attack rate calculation.

¶
https://www.cdc.gov/coronavirus/2019-ncov/if-you-are-sick/quarantine.html

** County A reported that 64% of their high school students were in-person learners. Using the date of specimen collection for cases and exposure date for contacts, the number of missed days of school was calculated, accounting for weekends and the holiday school closure. Patients isolated for 10 days and contacts quarantined for 14 days. The number of missed school days for patients and contacts was summed and multiplied by the percentage of in-person learners (64%) to arrive at the estimated number of lost in-person school days.

††
https://www.cdc.gov/coronavirus/2019-ncov/community/schools-childcare/indicators.html
